# Fodrin in Centrosomes: Implication of a Role of Fodrin in the Transport of Gamma-Tubulin Complex in Brain

**DOI:** 10.1371/journal.pone.0076613

**Published:** 2013-10-01

**Authors:** Sasidharan Shashikala, Rohith Kumar, Nisha E. Thomas, Dhanesh Sivadasan, Jackson James, Suparna Sengupta

**Affiliations:** 1 Cancer Research Programme III, Rajiv Gandhi Centre for Biotechnology, Thiruvanantha-puram, Kerala, India; 2 Neuro Stem Cell Biology, Rajiv Gandhi Centre for Biotechnology, Thiruvananthapuram, Kerala, India; Institute of Enzymology of the Hungarian Academy of Science, Hungary

## Abstract

Gamma-tubulin is the major protein involved in the nucleation of microtubules from centrosomes in eukaryotic cells. It is present in both cytoplasm and centrosome. However, before centrosome maturation prior to mitosis, gamma-tubulin concentration increases dramatically in the centrosome, the mechanism of which is not known. Earlier it was reported that cytoplasmic gamma-tubulin complex isolated from goat brain contains non-erythroid spectrin/fodrin. The major role of erythroid spectrin is to help in the membrane organisation and integrity. However, fodrin or non-erythroid spectrin has a distinct pattern of localisation in brain cells and evidently some special functions over its erythroid counterpart. In this study, we show that fodrin and γ-tubulin are present together in both the cytoplasm and centrosomes in all brain cells except differentiated neurons and astrocytes. Immunoprecipitation studies in purified centrosomes from brain tissue and brain cell lines confirm that fodrin and γ-tubulin interact with each other in centrosomes. Fodrin dissociates from centrosome just after the onset of mitosis, when the concentration of γ-tubulin attains a maximum at centrosomes. Further it is observed that the interaction between fodrin and γ-tubulin in the centrosome is dependent on actin as depolymerisation of microfilaments stops fodrin localization. Image analysis revealed that γ-tubulin concentration also decreased drastically in the centrosome under this condition. This indicates towards a role of fodrin as a regulatory transporter of γ-tubulin to the centrosomes for normal progression of mitosis.

## Introduction

Centrosomes in mammalian cells direct the nucleation of microtubules which are required for the motility and intracellular transportation of vesicles during interphase. During mitosis, centrosomes direct the formation of bipolar spindles [1] that are essential for the segregation of chromosomes. Localization of gamma tubulin complex at the centrosome contributes immensely to fulfil this role efficiently. γ-tubulin is present in the centrosome throughout the cell cycle. However, during centrosome maturation prior to mitosis, its concentration increases dramatically [2]. γ-tubulin is a highly conserved member of the tubulin family that occurs in the microtubule organising centres (MTOCs) [3]. It associates with other proteins to form two types of complexes [4], γ-TuSC and γ-TuRC. These complexes catalyse the nucleation of microtubules from MTOCs [5] although much less prominent, non-centrosomic nucleation of microtubules has also been reported [6], [7].

Gamma-tubulin small complex γ-TuSC, consisting of GCP2 (Gamma tubulin Complex Protein) and GCP3 along with γ-tubulin, is thought to be sufficient for the nucleation activity. GCPs 4, 5 and 6 associate with as many as seven γ-TuSC to form a larger ring complex γ-TuRC to give fidelity to the nucleation process in higher organisms [8]. A large amount of γ-TuRC exists as soluble cytoplasmic complexes [9]. Even though purified γ-TuRC from some systems have showed nucleation capacity, the complex when present in the cytoplasm, shows no nucleation activity, suggesting a probable control of nucleation capacity in the cytoplasm [10]. The transporting mechanism of the γ-TuRC from the cytoplasm to the centrosome is still not clear, though proteins like NEDD1 [11], ninein [12], AKAP450 [7] and pericentrin [13] which anchor γ-TuRC to centrosome have been identified.

Recently, MOZART 1 and MOZART 2 have been identified as proteins associated with the γ-TuRC in human cells [14], [15]. A recent report also showed the presence of non-erythroid spectrin in cytoplasmic γ-tubulin complexes in brain tissue [16].

Spectrin lines the intracellular side of the plasma membrane of many cell types. Spectrin is a cytoskeleton protein that forms pentagonal or hexagonal patterned scaffold which plays a pivotal role in the maintenance of cytoskeletal structure integrity of plasma membrane [17]. Spectrin may also play a role in maintaining the elasticity of the red cell membrane [18]. Fodrin/non-erythroid spectrin is a closely related tetrameric complex composed of two subunits, a 240 kDa (α) and 235 kDa (β), which is abundant in brain [19]. The αII subunit of fodrin contains an unusual domain that contains a binding site for calmodulin [20]. The phosphorylated form of the β subunit of both spectrins contains a binding site for syndein/ankyrin, a potential site for membrane attachment [21]. However, ankyrin independent mechanism of spectrin assembly has also been observed in Drosophila [22]. Other proteins f-actin and protein 4.1 attach to the ends of the bivalent brain spectrin/fodrin tetramer [23]. Some of these interactions points towards versatile functions of fodrin.

Centrosomal protein 4.1R is also an anchoring protein that establishes a bridge between the microtubules and the centrosomes. They participate in the dynamic interrelationships between the centrosome and the cytoarchitecture [24]. Spectrin and dynein is known to involve with early form of vesicles and aid in their transport to respective sites [25]. Fodrin is reported to associate with dynactin complex through its component protein centractin (ARP1), which provides a link to dynein and presumably other motors involved with intracellular transport [26], [27]. Other than its regular function of maintaining cell architecture, fodrin also plays a role in axonal transport and movement of vesicles and organelles [28]–[30].

In this report, we have characterized the association of non-erythroid spectrin/fodrin with γ-tubulin in different types of brain cells. Since centrosomes are the major centre for γ-tubulin function, we checked the association in centrosomes and other subcellular organelles. We have found that fodrin is associated with γ-tubulin in both the cytoplasm and centrosomes in most of the brain cells and the association in centrosome is actin dependent. However, fodrin dissociates from the centrosome during mitosis.

## Materials and Methods

### Materials

PMSF (Phenyl Methyl Sulfonyl Flouride), DTT (Di Thio Treitol), Aprotinin, leupeptin, pepstatin, urea, heparin, FGF, EGF (Epidermal Growth Factor), Aphidicolin and Nocodazole were from Sigma, USA. F12 and DMEM media were from Gibco, USA. N2 supplement was procured from Invitrogen, USA. FBS was purchased from Pan Biotech, Germany. All other reagents were of reagent grade.

### Antibodies

Primary antibodies: polyclonal rabbit and monoclonal mouse Anti alpha fodrin, rat anti YOL1 Tub marker, rabbit-polyclonal Anti Ninein, mouse monoclonal Anti Pericentrin and mouse monoclonal Anti Centrin were from Abcam, UK; mouse monoclonal GFAP, rabbit polyclonal and monoclonal mouse MAP2 and rabbit anti-γ-tubulin were from Sigma, USA.

Secondary antibodies: Anti mouse alexa 488, anti rabbit alexa 568, anti rat alexa 633 were from Molecular Probes, USA.

### Cell lines

IMR32 (nueuroblastoma), U373 (glioblastoma), Hela (cervical cancer) were from ATCC.

### Methods

#### Ethics Statement

All animal experiments were carried out after getting approval from the Institutional Animal Ethics Committee, Rajiv Gandhi Centre for Biotechnology (Proposal no. IAEC/153/SUP/2012), Govt. of India. All the guidelines for animal maintenance, handling and euthanasia were followed.

### Cell culture

IMR32, U373 and Hela cells were cultured in DMEM supplemented with 5% fetal calf serum and antibiotics.

### Primary cell culture

Mouse E18 neural progenitors were generated according to [31] and maintained in FGF2 and EGF containing medium. Neuronal proliferation medium consisted of Dulbecco's modified Eagle's medium-F12 supplemented with 1% N2 supplement, Heparin (2 µg/mL) and 20 ng/mL FGF2. In differentiating condition the cells were also supplemented with 1% FBS.

### Centrosome purification

Centrosomes were purified following the protocol of Mitchison and Kirshner [32]. For isolation of centrosomes from tissue, a single cell suspension was derived from brain tissue and subjected to nocodazole and cytochalasin treatment. Cell lines were given the same treatment by addition of culture medium (DMEM with 5% FBS) containing nocodazole and cytochalasin B. After 90 mins of treatment, the cells were washed, collected and lysed in non-ionic lysis buffer (1 mM HEPES, 0.5% NP40, 0.5 mM MgCl_2_, 0.1% β-ME,1 mM PMSF, 1 µg/mL aprotinin and 1 µg/mL leupeptin). Centrosomes were isolated by loading the lysate on a 70% sucrose cushion followed by centrifugation at 1,00,000X g for 2 hrs. The concentrated centrosomes were purified on a 20–60% sucrose gradient. The purity was checked by SDS-PAGE and western blot using centrosome specific proteins. The isolated fraction containing centrosomes was diluted in 1X PE (100 mM PIPES, 1 mM EDTA) and the centrosomes were pelleted down. They were then reconstituted in 1X PE (100 µl) and subjected to western blot.

### Immunoprecipitation

The centrosome fraction purified by sucrose gradient was further subjected to immuno pull downs by anti α-fodrin, anti-γ-tubulin, anti-ninein antibodies coupled to Protein A Agarose. The pulled down fractions were then checked with all the above antibodies and anti centrin/anti pericentrin antibodies to confirm the purity of isolated centrosomes and the interaction of these proteins in centrosomes.

### Western blot

All the sucrose gradient fractions from goat brain tissue, brain specific cell lines and immuno precipitation samples of purified centrosomes from brain tissue were lysed with non-ionic lysis buffer, pelleted and reconstituted with centrosome extraction buffer (Trisbase 20 mM, Na.EDTA (2 mM), sodium deoxy cholate (0.5%), NP-40 (0.5%), SDS (0.1%), Urea (8 M) and kept for 10 mins at 4°C. 40–50 µg of this lysate was immunoblotted with appropriate dilutions of antibody, followed by incubation with horse radish peroxidise conjugated secondary antibody. The protein bands were observed by using enhanced chemiluminescence detection system. (GE Amersham).

### Immunocytochemistry and confocal imaging

Standard protocols were applied for immunofluorescence of cells grown on coverslips or glass bottom dishes (BD Biosciences, USA). After attaining the required conditions, they were fixed in chilled methanol at −20°C for 10 minutes. The primary antibodies were used in 1∶200 dilution and secondary antibodies were used in 1∶500 dilution in PBST containing 5% BSA. Confocal fluorescence imaging was done in either Nikon Eclipse Ti(AIR), Japan or Leica TCS SP2, Germany microscope.

### Cell synchronisation

IMR32 cells were synchronised by incubation with aphidicolin (2 µg/mL) for 18 hrs. The cells were then washed and released in drug free DMEM. Subsequently, they were allowed to enter into the cell cycle and fixed at different time points using −20°C chilled methanol to obtain different stages of mitosis.

### Depolymerization of actin and microtubule cytoskeleton

IMR32 cells were given Nocodazole (3.3 µM, 2 hrs) or Cytochalasin B treatment (1 µM, 1 hr) for inhibiting microtubule and actin polymers respectively. The cells were then immunostained with γ-tubulin, tubulin or actin primary antibody followed by respective Alexa fluor conjugated secondary antibodies.

### Fluorescence Intensity Measurements

Archived images of 16 bits were analysed for γ-tubulin/α-fodrin fluorescence intensity in IMR32 cells for centrosomes of interphase and mitotic cells. Both control and Cytochalasin B treatment groups were subjected to similar conditions of Laser Power and voltage. Imaging was done on NIKON ECLIPSE Ti microscope attached with a Nikon A1R confocal unit. Nikon imaging software AR4.00.04 was calibrated using a micrometer for 60X objective. Images were then integrated into the NIS elements software's automated measurement tool for ROI analysis. Average intensity values for a region of interest in pixel (Mean Intensity ×ROI Area covering the centrosomes) were obtained for centrosomes of interphase and mitotic cells both in control group and after cytochalasin treatment. Each ROI was selected after subtraction of background γ-tubulin/α-fodrin fluorescence intensity values of cytoplasm for better visualisation of centrosomes. At least 15 centrosomes per treatment group were examined, and each experiment was run in triplicate.

## Results

γ-TuRC is the major nucleating agent of microtubules in the cell. It is present in both cytoplasm and centrosome. Non-erythroid spectrin/fodrin is associated with cytoplasmic γ-TuRC purified from goat brains [16]. To further characterize and to know the significance of this association, we investigated the localization of fodrin and γ-tubulin in brain cells under different conditions.

### Co-localization of fodrin and γ-tubulin in brain

**Figure 1:**
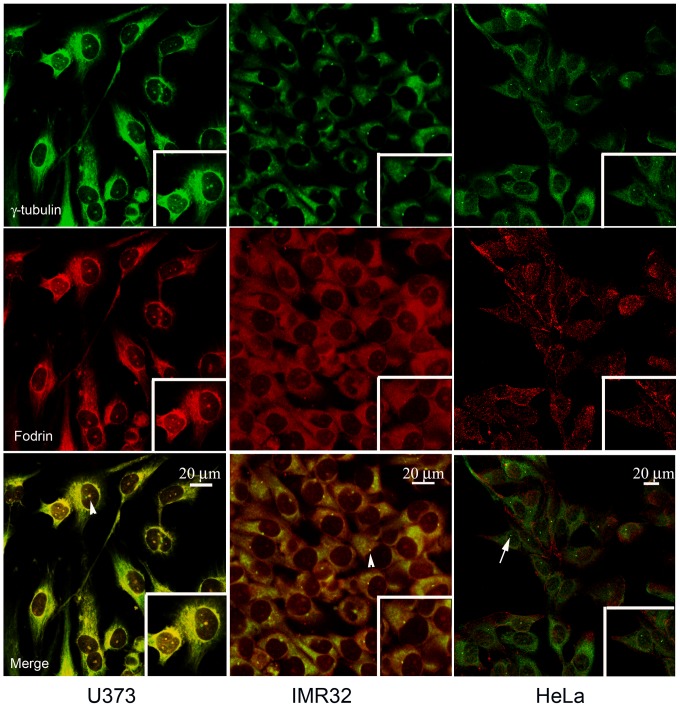
Co-localization of γ-tubulin and fodrin in neuroblastoma and glioblastoma cell lines. U373 and IMR32 cell lines were immunostained with antibodies in 1∶200 dilution against α fodrin (red) and γ-tubulin (green). Secondary antibodies were used in 1∶500 dilution. The magnified image of a single cell where co-localization occurs is shown as inset. Arrowheads mark co-localization and arrows illustrate absence of co-localization. Cells were maintained in DMEM supplemented with 5% fetal calf serum and antibiotics. HeLa cells were used as negative control.

Neuronal and glial cells constitute the two major types of cells in mammalian brain. Neuronal cells are actively involved in the axonal transport of neurotransmitters [33] while the glial cells are involved in the axonal transport of nutrient vesicles and other hormones [34]. To check if fodrin is co-localized with γ-tubulin in all types of brain cells, immunocytochemistry has been performed on glioblastoma cell line U373 and neuroblastoma cell line IMR32. HeLa cells, which are of cervical origin, have been used as control. Specificity of the fodrin antibodies was initially checked by western blot (Figure S1). Cytoplasmic and centrosomal co-localization is observed in almost 100% of the cells in focus for both U373 and IMR32 cell lines but not in HeLa cells (Figure 1). This shows that fodrin is co-localized with γ-tubulin in cell lines derived from brain. These results are in accordance with an earlier observation that this co-localization is found only in brain [16].

**Figure 2:**
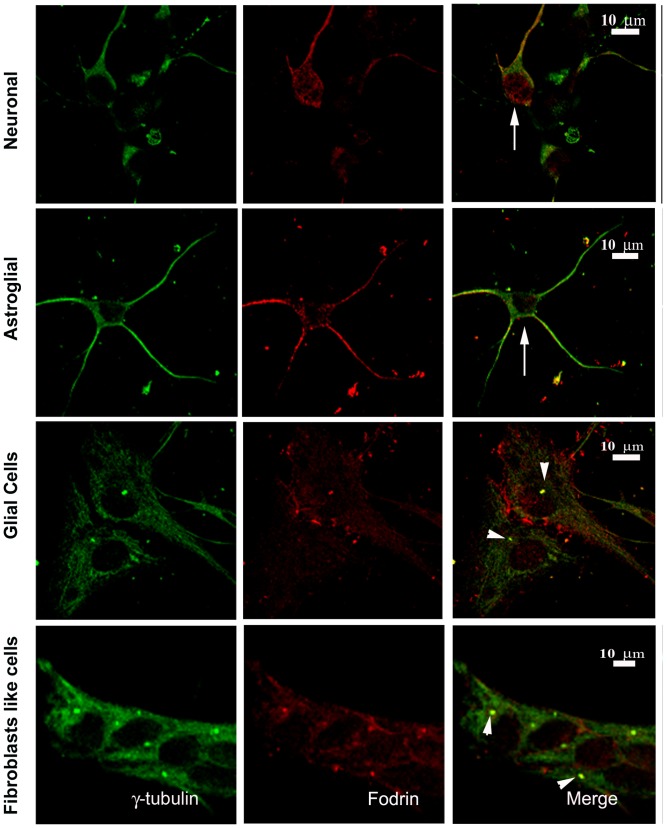
Co-localization of γ-tubulin and fodrin in primary cells. Cells were isolated from 18 days old mouse embryonic brain and cultured in appropriate media as described in the methods section. Cells were imaged after immunostaining with α-fodrin (red) and γ-tubulin (green) as described above. Arrowheads indicate co-localization and arrows illustrate absence of co-localization.

Earlier, immunohistochemical analysis detected fodrin in mammalian brain tissue sections [16]. To rule out the possibility that the association pattern is specific to cancer cell lines, we have further checked the association of fodrin with γ-tubulin in a primary culture of cells extracted from 18 day (E18) old mouse embryonic brain. Immunocytochemistry using γ-tubulin and fodrin antibodies showed the co-localization of fodrin and γ-tubulin in both centrosome and cytoplasm of glial and fibroblast like cells. However, labelling of γ-tubulin and fodrin was not observed in the centrosomes of neuronal cells and astroglial cells showing that they were not present in the centrosomes (Figure 2). Antibodies against glial marker GFAP (Glial Fibrillary Acidic Protein) and matured neuronal marker MAP2 (Microtubule Associated Protein) were used along with fodrin in a parallel culture (Figure S2) to confirm that indeed fodrin was present in the centrosome of glial cells but absent in the centrosome of matured neuronal cells. It has been earlier reported that neuronal progenitor cells lack centrosomal γ-tubulin after differentiation when they form synaptic connections [35]. It has also been proposed that mature astrocytes are primarily postmitotic [36]. Thus the absence of γ-tubulin and fodrin in the centrosomes of astrocytes and differentiated neurons shows that their presence is not required in the centrosomes of non-dividing cells.

**Figure 3:**
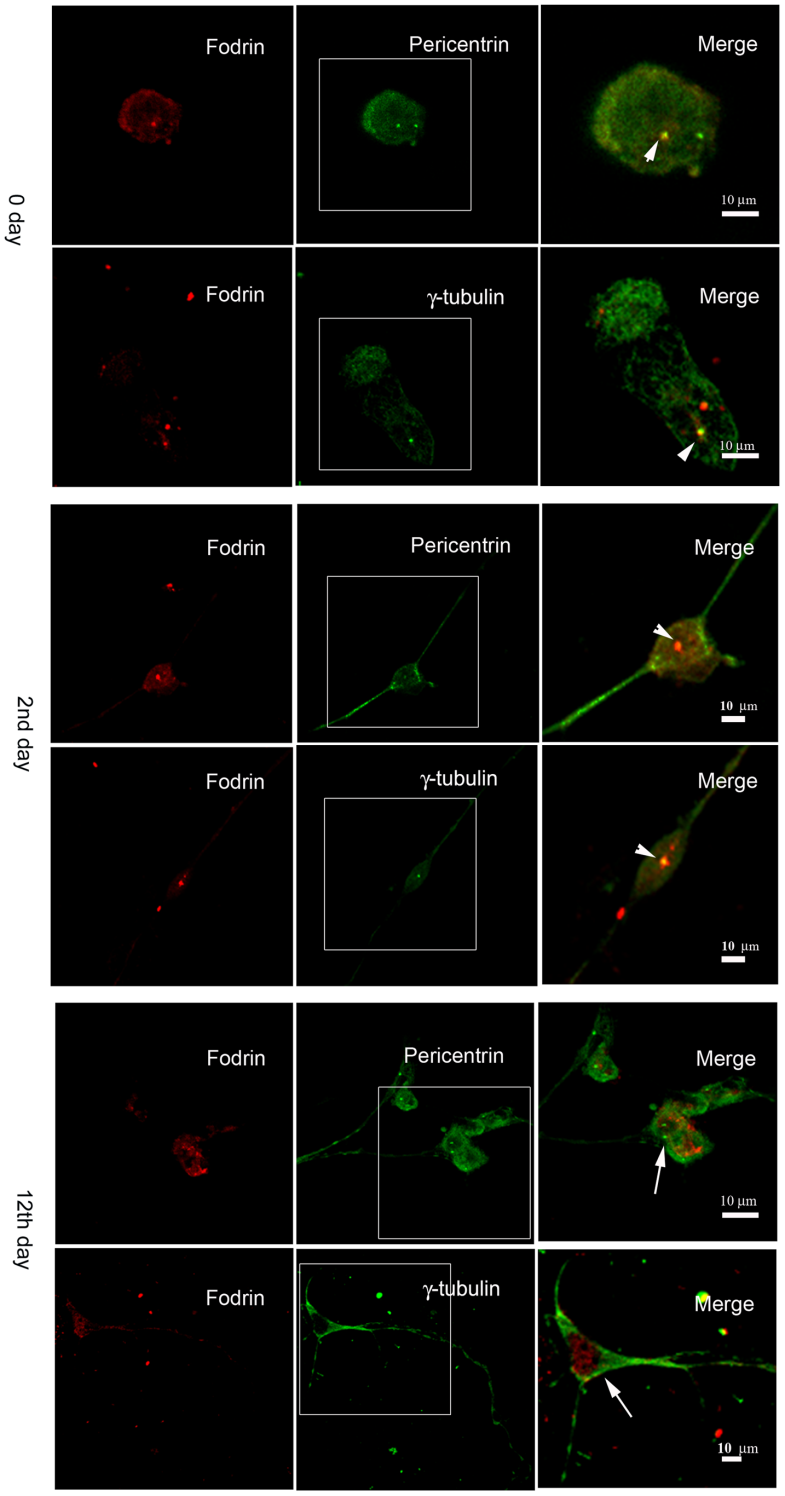
Co-localization α-fodrin and γ-tubulin in primary neurons at different stages. Cells were isolated from E18 mouse embryo and were maintained in neuronal cell enrichment media. Panels show fodrin co-localization with either pericentrin (centrosomal marker) or γ-tubulin in undifferentiated (0 day), early (2^nd^ day) and late stages of differentiation (12^th^ day). Immunostaining was done with α-fodrin (red), γ-tubulin (green) and pericentrin (green) as described above. Area of the cells in the boxes is magnified and shown in merge. Arrowheads mark co-localization and arrows indicate the absence of co-localization.

We next checked the status of co-localization of these two proteins in neuronal cells at different stages of differentiation in a neuronal enrichment media containing FGF2 (Fibroblast Growth Factor). Undifferentiated proliferative cells isolated from E18 mouse embryo showed the presence of centrosomal γ-tubulin and it co-localized with α-fodrin in both cytoplasm and centrosome (Figure 3). Similar pattern of co-localization was observed in case of neuronal cells at early differentiating stage i.e., two days after putting them in the differentiating media. Fully differentiated neuronal cells (after 12 days of differentiation), however, did not show the presence of centrosomal γ-tubulin and fodrin. In a parallel culture, MAP2 was used along with fodrin to show that indeed mature neurons lack fodrin in their centrosomes (Figure S3). Since fodrin is a cytoskeletal component, these results indicate that it is found in the centrosome only when γ-tubulin is present.

### Co-localization in purified centrosomes

Centrosomes are the microtubule organizing centres in a eukaryotic cell from where γ-TuRC nucleates microtubules. Our immunocytochemistry results showed that fodrin is present along with γ-tubulin in the centrosomes. We thus checked the co-occurrence of γ-tubulin and fodrin in purified centrosomes.

**Figure 4:**
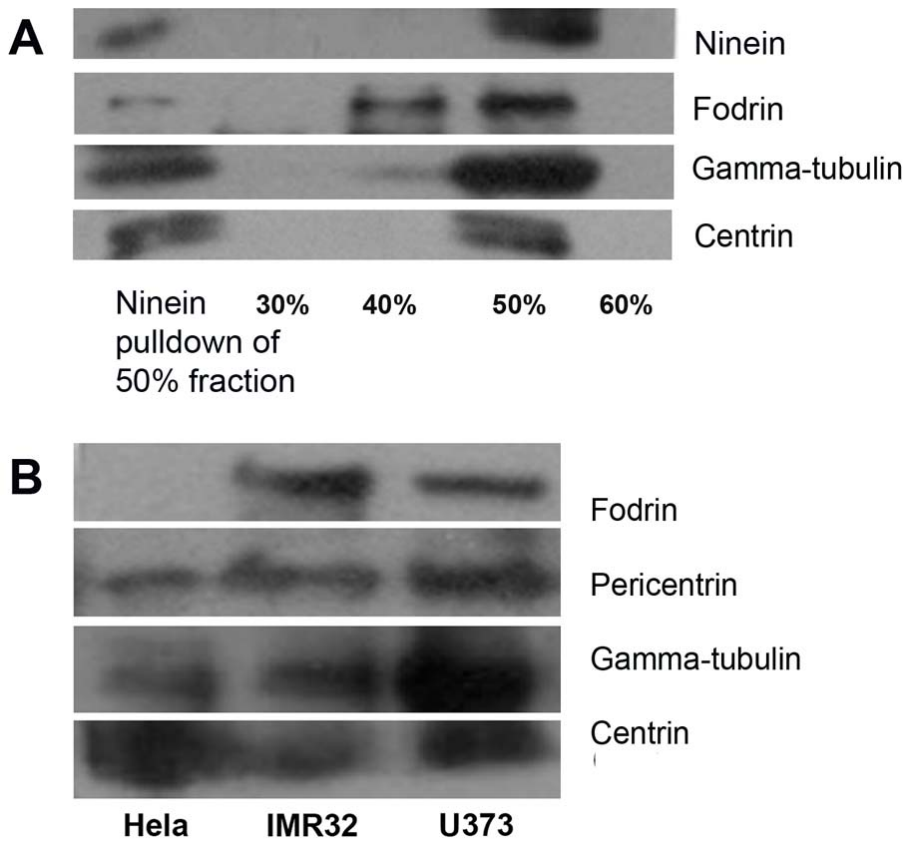
Fodrin and γ-tubulin interaction in purified centrosomes. A: Different fractions of sucrose density gradient separation of isolated centrosomes from goat brain tissue were immunoblotted with Ninein, γ-tubulin and Centrin in 1∶1000 dilution and α-fodrin in 1∶500 dilution. Secondary antibodies were used in 1∶2000 dilution. The 50% fraction was further immunoprecipitated using anti-Ninein antibody and was probed as above (leftmost lane) B: Centrosomes purified from HeLa, IMR32 and U373 cell lines were immunoblotted and probed for the presence of α-fodrin, pericentrin, γ-tubulin and centrin as described above. Pericentrin antibody was used in 1∶1000 dilution.

Centrosomes were purified from goat brain tissue. The 50% fraction of a sucrose density gradient separation of centrosomal lysate was found to contain the maximum amounts of centrosomal marker proteins ninein and centrin (Figure 4A). This fraction also showed the presence of γ-tubulin and fodrin. To further confirm that spectrin was not present as impurity, the 50% sucrose gradient fraction was reconstituted and subjected to immuno pulldown with an antibody against ninein, a centrosomal marker protein, to get highly pure centrosomes. Western blotting of this eluent showed the presence of fodrin along with γ-tubulin and other centrosomal constituents ninein and centrin. This confirms that fodrin is present in pure centrosomes along with γ-tubulin. Further, immunoprecipitation with anti γ-tubulin or anti α-fodrin antibodies could pull down fodrin, γ-tubulin, ninein and centrin (Figure S4). This confirms that fodrin, γ-tubulin and ninein interact with each other in centrosomes.

Centrosomes were also purified from IMR32 and U373 cell lines. These centrosomes also showed the presence of fodrin along with γ-tubulin and other centrosomal markers while centrosomes isolated from HeLa (used as a control cell line not originating from brain) did not show the presence of fodrin (Figure 4B). This confirms that γ-tubulin interacts with fodrin in centrosomes of neuroblastoma and glioblastoma cell lines.

Recently, non centrosomal nucleation have been reported in some systems [37]. Recent data have indicated γ-tubulin dependent formation of microtubules from the Golgi apparatus in human epithelial cells and hepatocytes [6], [38]. To check if the co-localization of fodrin with γ-tubulin is found in the Golgi Apparatus (GA) of neuronal cells, we have done immunofluorescence studies in IMR32 cells using an antibody against a GA marker protein Golgin GM130. However, staining with antibodies against fodrin and γ-tubulin have not shown localization of any of these proteins in the golgi apparatus as neither of them co-localize with Golgin GM130 (Figure S5). This indicates that in these cells, fodrin's association along with γ-tubulin complex is specifically targeted for functions at centrosomes.

### Lack of centrosomal co-localization of γ-tubulin and fodrin in mitosis

**Figure 5:**
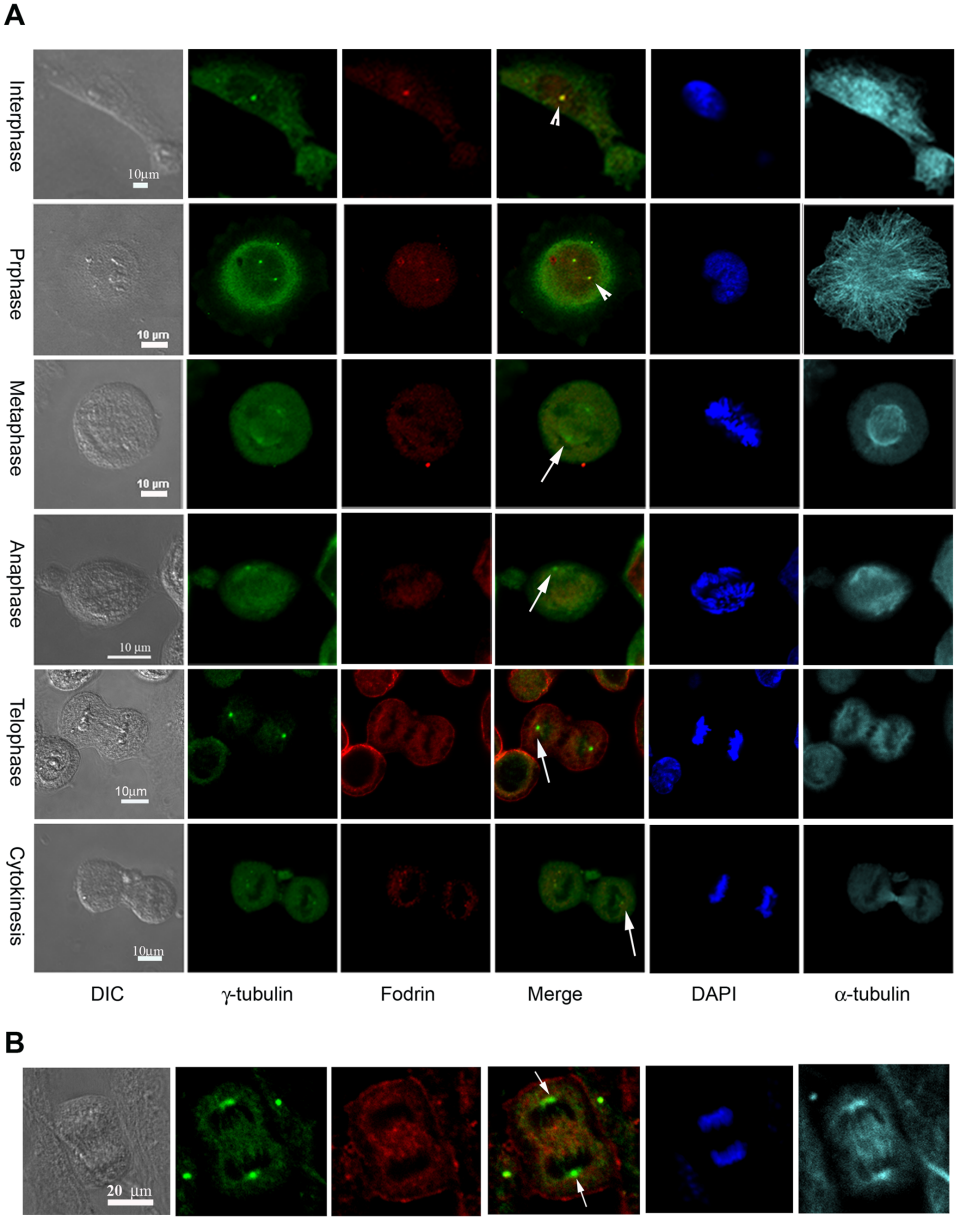
Co-localization of γ-tubulin and fodrin at different stages of mitosis. A: IMR 32 cells were synchronised by aphidicollin treatment and then were released to enter into the cell cycle. Cells were then imaged at different stages of mitosis. α-fodrin (red), γ-tubulin (green), α-tubulin (cyan) and DNA (blue). Co-localization in the centrosome is shown by arrowheads and the absence is indicated by arrows. B: Mitotic cells in primary cell culture isolated from 18 days old mouse embryonic brain and cultured in appropriate media was immunolabelled with α-fodrin (red), γ-tubulin (green) and α-tubulin (cyan). Arrowheads indicate co-localization and arrows illustrate absence of co-localization. Antibody staining was done as in figure 1.

**Figure 6:**
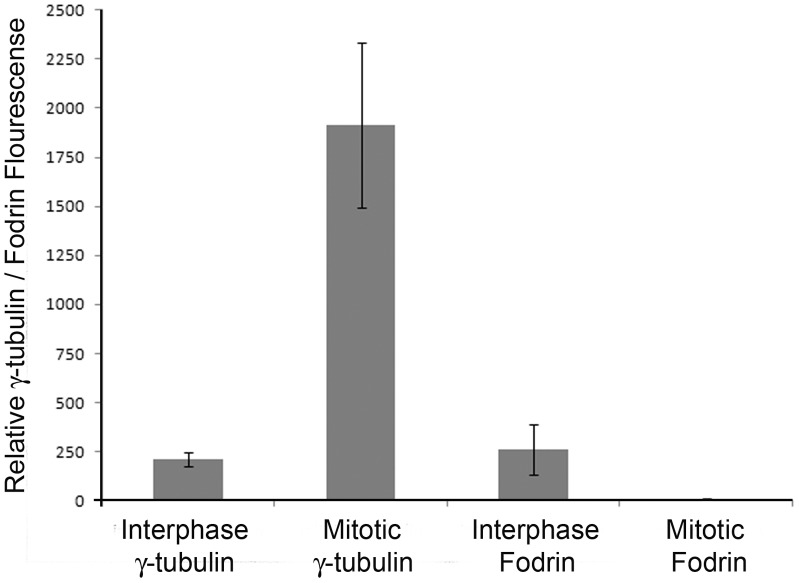
Quantitation of γ-tubulin and fodrin in interphase and mitotic cells: IMR 32 cells from the above experiment were analyzed for γ-tubulin or fodrin content in green chanel and red channel respectively. The ROI was selected to cover the whole centrosome in different cells and the total fluorescence intensity (pixels) was obtained by multiplying the mean intensity with centrosome area. The background was selected similarily from the cytoplasmic area and subtracted from the centrosomal intensity. The average of thus calculated intensities was determined from 21 centrosomes and plotted. Standard deviation was calculated from three experiments.

During the onset of mitosis there is an evident increase in the localization of γ-tubulin in the centrosomes along with other proteins such as centrosomin and cdc25b [39], [40]. Presumably these proteins are transported and their transport appears to happen in a regulated manner. In an initial experiment, we observed that in mitotic cells fodrin co-localization with γ-tubulin was absent in the centrosomes. To check this in detail, co-localization pattern of fodrin and γ-tubulin in the centrosome was studied in IMR32 cells at different stages of mitosis. The cells were synchronised by aphidicolin and after 18 hours of treatment, they were released and imaged at different time points. Untreated interphase cells were used as controls. During interphase and prophase, fodrin was found to be co-localized with γ-tubulin in centrosomes, but by metaphase and throughout anaphase, telophase and cytokinesis, the presence of fodrin at centrosomes clearly diminished (Figure 5A). A mitotic cell from a primary culture of mouse embryonic brain also showed the absence of spectrin in the centrosome, even though γ-tubulin was present (Figure 5B). Quantitation of fodrin and γ-tubulin in the interphase and metaphase of IMR32 cells showed that while the γ-tubulin concentration increased 9 fold, the concentration of fodrin decreased almost 60 fold when the cells entered into mitosis (Figure 6). From these observations, it is implied that fodrin is not required in the centrosome during mitosis and probably it is dissociated or degraded after the entry of the cell into mitosis.

### Dependence on actin cytoskeleton

**Figure 7:**
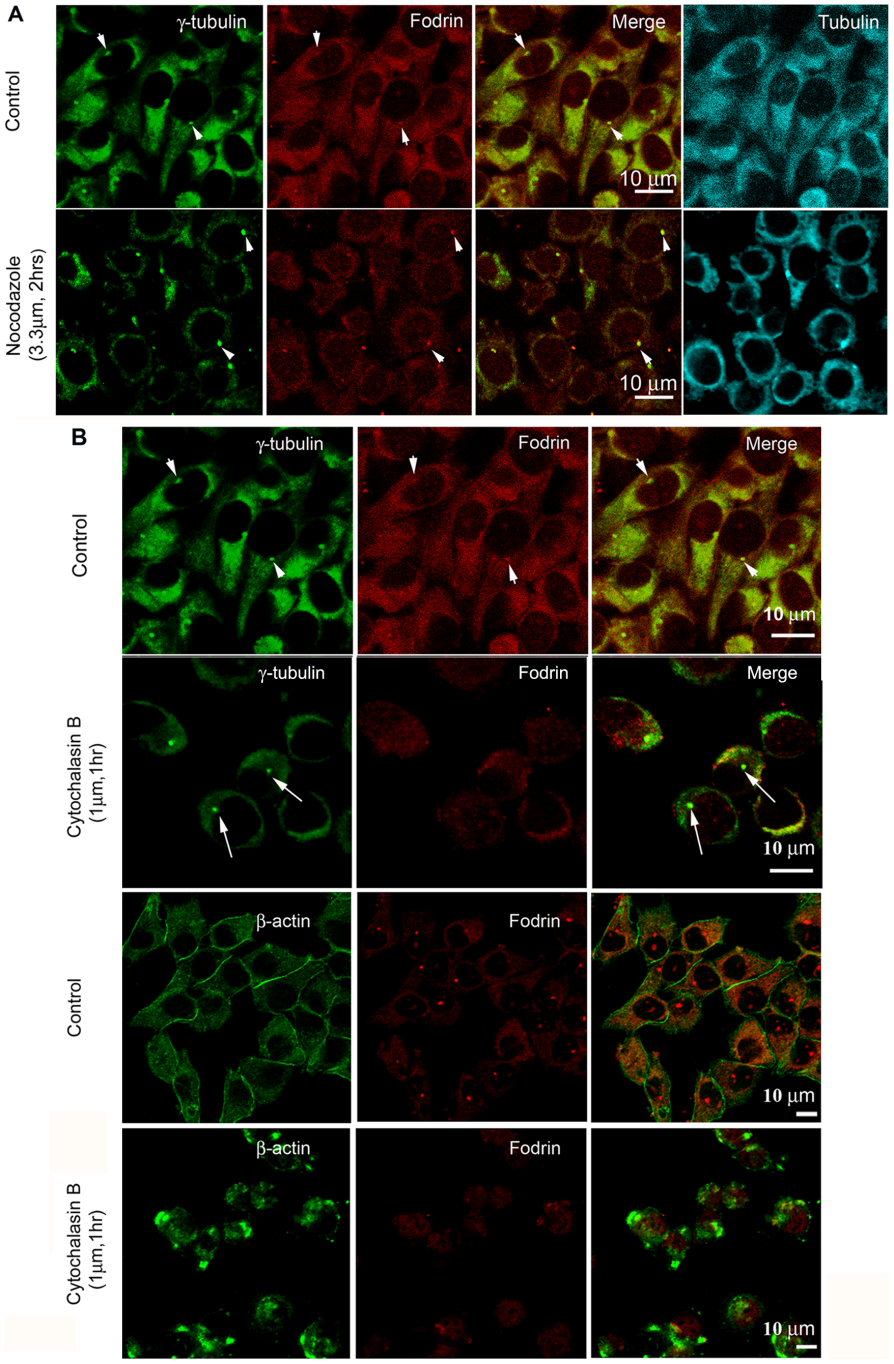
Effect of microtubule and actin cytoskeleton on the centrosomal co-localization of fodrin and γ-tubulin. A. IMR32 cells were treated with Nocodazole (3.3 µM) for 2 hrs and then stained with α-fodrin (red), γ-tubulin (green) and α- tubulin (cyan). (B) IMR32 cells were treated with Cytochalasin B (1 µM) for 1 hr and then stained with either α-fodrin (red) and γ-tubulin (green) or with α-fodrin (red) and β actin (green). Control cell panels for γ-tubulin and fodrin staining have been reproduced from 7A as the experiments were done together. Co-localization is indicated by arrowheads and the absence of co-localization is shown by arrows. Primary antibodies were used in 1∶200 dilution and secondary antibodies were in 1∶500 dilution.

Both fodrin and γ-tubulin are individually known to interact with tubulin and actin [41]–[47]. To unravel whether this centrosomal interaction between γ-tubulin and fodrin was actin or microtubule dependent, the IMR32 cells were treated with microtubule depolymerising and actin depolymerizing agents Nocodazole and Cytochalasin B [48], [49] respectively. Treatment of 3.3 µM Nocodazole for 2 hrs did not have any effect on the co-localization pattern of fodrin and γ-tubulin both at the centrosomal and cytoplasmic levels (Figure 7A, merged panel) even though under this condition, microtubule cytoskeleton depolymerized (Figure 7A, right panels). However, upon treatment with 1 µM of Cytochalasin B for 1 hr, the centrosomal co-localization of fodrin and γ-tubulin was not observed as fodrin was lost from the centrosome (Figure 7B, top panels). Labelling with an antibody against actin showed that under this condition, actin cytoskeleton depolymerized (Figure 7B, bottom panels). The results point out that fodrin localization in the centrosome is actin dependent.

**Figure 8:**
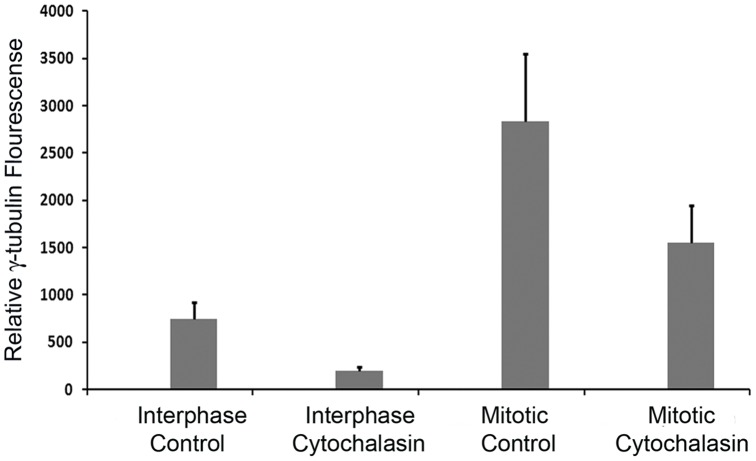
Measurement of centrosomal γ-tubulin fluorescence after depolymerization of actin cytoskeleton: IMR 32 cells from the above experiment were analyzed for γ-tubulin content in the centrosome as described in Figure 6. The average of thus calculated intensities was determined from 15 centrosomes and plotted. Standard deviation was calculated from three experiments.

To know the effect of this loss of fodrin on centrosomal γ-tubulin, the amount of γ-tubulin was analysed by measuring the fluorescence intensity in control and cytochalasin treated centrosomes. The average intensity of γ-tubulin fluorescence calculated over the whole centrosome area of Cytochalasin B treatment group showed a decrease of 74% from the control group's average fluorescence intensity of γ-tubulin in interphase cells. The centrosome intensity measurements of mitotic cells treated with Cytochalasin B showed a decrease of 45% as compared to the mitotic control group (Figure 8). As expected, γ-tubulin intensity increased many fold in both the control and treatment groups during mitosis as compared to interphase centrosomes. Thus the loss of fodrin by actin cytoskeleton depolymerisation was accompanied by a reduction in the γ-tubulin amount in the centrosome of IMR32 cells.

## Discussion

Classically, spectrin is known to maintain the structural integrity of the cell membrane, and is involved in controlling the lateral mobility of integral membrane proteins (for review, see [50]. Spectrin is also known to be cleaved during apoptosis [51] and is involved in TGF β signalling pathway [52]. Non erythroid spectrin/fodrin, which is abundant in brain, is known for some additional functions. It plays a role in the axonal transport and movement of organelles [28], [30]. αII-Spectrin, found mostly in brain tissue, participates in DNA interstrand repair by binding to damaged DNA and helps in the recruitment of repair proteins [53], [54]. Recently non erythroid spectrin has been implicated in cell cycle regulation through regulation of the expression of p21 [55]. The findings that fodrin is associated with γ-tubulin in brain [16], thus, raises intriguing questions.

Centrosomes are the major nucleating centres in eukaryotic cells from where γ-tubulin, in association with other proteins (γ-TuRC), nucleates microtubules. Earlier reports have shown that all the components of γ-TuRC are present both in the cytoplasm and the centrosome. However, it is not clear about the control mechanism that resists nucleation from the cytoplasm and how the ring complex gets transported to the centrosome. While members of this complex like GCP2 and GCP3 are required for nucleation, the other members like GCP4, GCP5 and GCP6 are required in higher organisms for giving perfection to the nucleation process [8]. Earlier it has been found that fodrin is a part of the cytoplasmic γ-TuRC [16]. Our observation here that fodrin is present in the centrosomes indicates that fodrin also has a role in the γ-tubulin mediated functions from centrosomes in brain cells. We have, however, not observed either γ-tubulin or fodrin in the centrosomes of differentiated neurons isolated from mouse embryonic brain, even though they were co-localized in the centrosome of undifferentiated neurons and neurons in the early differentiated stage (Figure 3). This is in accordance with earlier observation where it was reported that γ-tubulin is present in the centrosomes of neurons in their early differentiation stage to provide sufficient microtubule polymer for rapid axonal growth, but not in the centrosomes of neurons which has formed functional synaptic connections [35]. In neurons, microtubules are nucleated in the centrosomes, but after nucleation they get detached from the centrosomes when γ-tubulin is lost. Our observation in primary cells thus shows that fodrin's presence in the centrosome is connected with γ-tubulin. Non-centrosomal nucleation has been reported in some cases. Plant γ-tubulin forms membrane associated complexes and nucleates microtubules from the cortex [42]. Golgi associated nucleation involving γ-TuRC have been reported in hepatocytes and human pigment epithelial cells [6], [7]. We have not observed substantial labelling of γ-tubulin or fodrin in Golgi bodies as there was no co-localization of the golgi specific protein Golgin GM130 with either of them in the neuroblastomal cell line IMR32 indicating that Golgi associated nucleation probably does not happen in these cells. It is reported that a different isoform of spectrin, spectrinIII is associated with golgi bodies [56], [57].

The co-localization of fodrin with γ-tubulin in the centrosome was found to be dependent on the actin cytoskeleton (Figure 7) and fodrin depletion by actin depolymerisation negatively regulated γ-tubulin. Interaction of fodrin with the actin cytoskeleton is long known [58], [59]. The centrosomal protein 4.1, which plays a role in microtubule anchoring to centrosomes, binds to fodrin [24]. It is thus possible and needs further work to confirm whether gamma tubulin complex gets transported to the centrosomes using actin spectrin network where protein 4.1 acts like a bridge.

There exists clear evidence that γ-tubulin concentration increases dramatically before the onset of mitosis. Our studies in neuronal cells during the course of mitosis showed that after prophase, localization of fodrin at centrosomes drastically diminished. This shows that after the transport of γ-TuRC, when mitosis starts, fodrin dissociates from γ-tubulin. This observation points out to an interesting possibility that fodrin has the role of a regulatory transporter in the accumulation of γ-tubulin at centrosome. In absence of fodrin after prophase, γ-tubulin nucleates spindle microtubules from the centrosome to proceed into mitosis. This assumption gets further support from the analysis of γ-tubulin amount in the centrosome of interphase and mitotic cells after depolymerisation of actin cytoskeleton. Depolymerization of actin polymers by cytochalasin treatment removed fodrin in the centrosome. In interphase cells, this removal of fodrin caused a 74% reduction of γ-tubulin in the centrosome, whereas in mitotic cells, where centrosomal γ-tubulin concentration is substantially high due to premitotic transport from the cytoplasm, loss of fodrin caused 45% reduction of γ-tubulin (Figure 8).

Calpains are a class of proteolytic enzymes which are known to play important role in apoptosis and in the regulation of cell cycle [60]–[62] in a calcium dependent manner. m-calpain is known to degrade fodrin [63], [64]. Studies on calpains suggest that their concentration increases during mitosis. Down regulation of m-calpain, but not μ-calpain, arrested cells at prometaphase, as they failed to align their chromosomes at the spindle equator. The down regulation of calpain expression was also found to activate the spindle assembly checkpoint [62]. It has been suggested that calpain activity has a role in the polar ejection force for alignment of chromosomes in metaphase as when calpain activity was inhibited, cells had monopolar spindles and chromosomes were clustered near the centrosomes. Thus it was concluded that activation of m-calpain during mitosis is a prerequisite for cells to establish the alignment of chromosomes by regulating some molecules that generate polar ejection force. It is thus highly probable that at least in brain cells, after the onset of mitosis when sufficient γ-tubulin has been transported to the centrosome, calpains cleave α-fodrin off its association from γ-tubulin leading to proper progression of mitosis.

**Figure 9:**
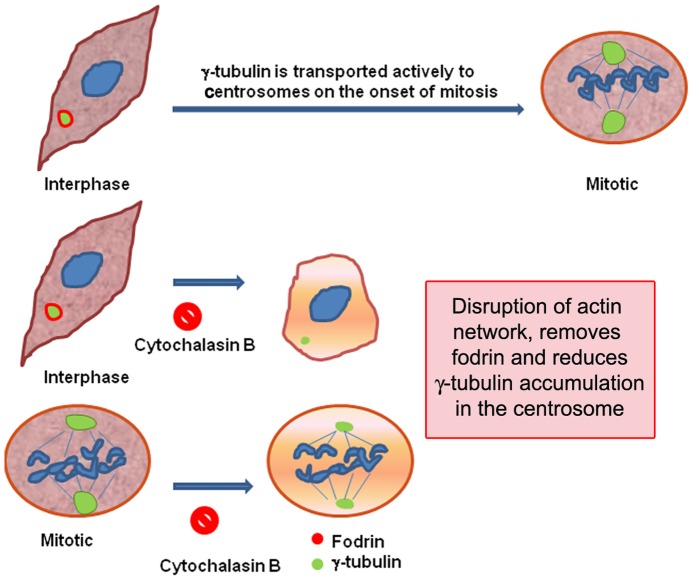
A model showing the putative role of fodrin in the transport of γ-tubulin to the centrosome.

Thus our studies implicate that fodrin associates with γ-tubulin in an actin dependant manner to aid in its regulated transport to centrosome for microtubule nucleation and mitosis, but dissociates from it as the cell progresses into mitosis (A scheme is shown in Figure 9).

## Supporting Information

Figure S1 **Purified γ-TuRC was treated with antibodies against α-fodrin at 1∶500 dilution and developed by respective secondary antibodies.**
(TIF)

Figure S2 **Localization pattern of MAP2 (mature neuronal marker), GFAP (glial marker) and fodrin in primary cells.** Cells were isolated from 18 days old mouse embryonic brain and cultured in appropriate media as described in the methods section. Cells were imaged after immunostaining with A: GFAP (green), MAP2 (red); B: MAP2 (green), α-fodrin (red) for mature neurons and C: GFAP (green), α-fodrin (red) for glial cells. DAPI (blue) was used for nuclear staining.
(TIF)

Figure S3 **Localization of α-fodrin and MAP2 in primary neurons at different stages.** Cells were isolated from E18 mouse embryo and were maintained in neuronal cell enrichment media. Panels show fodrin and MAP2 localization in undifferentiated (0 day), early (2^nd^ day) and late stages of differentiation (12^th^ day). Immunostaining was done with α-fodrin (red) and MAP2 (green). Area of the cells in the boxes is magnified and shown in merge. Arrowheads mark the presence of fodrin on 0 day and 2^nd^ day.
(TIF)

Figure S4 **Immunoprecipitation of centrosome fraction: The centrosome fraction (50%) purified by sucrose density gradient was immunoprecipitated with anti ninein, anti γ-tubulin and anti α-fodrin antibodies.** Immuno pulldown with PAg and anti Bid antibody were used as negative controls. Western blot was performed with antibodies against ninein, γ-tubulin and centrin in 1∶1000 dilution and α-fodrin in 1∶500 dilution.
(TIF)

Figure S5 **Localization of γ-tubulin or fodrin in golgi apparatus.** IMR32 cells were immunostained for A: DAPI, tubulin (cyan), γ-tubulin (red) and golgi matrix protein GM130 (green); B: DAPI, tubulin (cyan), fodrin (red) and golgi matrix protein GM130 (green). Primary and secondary antibodies were used in 1∶200 and 1∶500 respectively. Cells were maintained in DMEM containing 5% FBS with antibiotics.
(TIF)
