# Supplementary figures and images for: Fodrin in Centrosomes: Implication of a Role of Fodrin in the Transport of Gamma-Tubulin Complex in Brain
Source: PLoS One. 2013 Oct 1;8(10):e76613. doi: 10.1371/journal.pone.0076613 (PMC3788121; doi:10.1371/journal.pone.0076613)

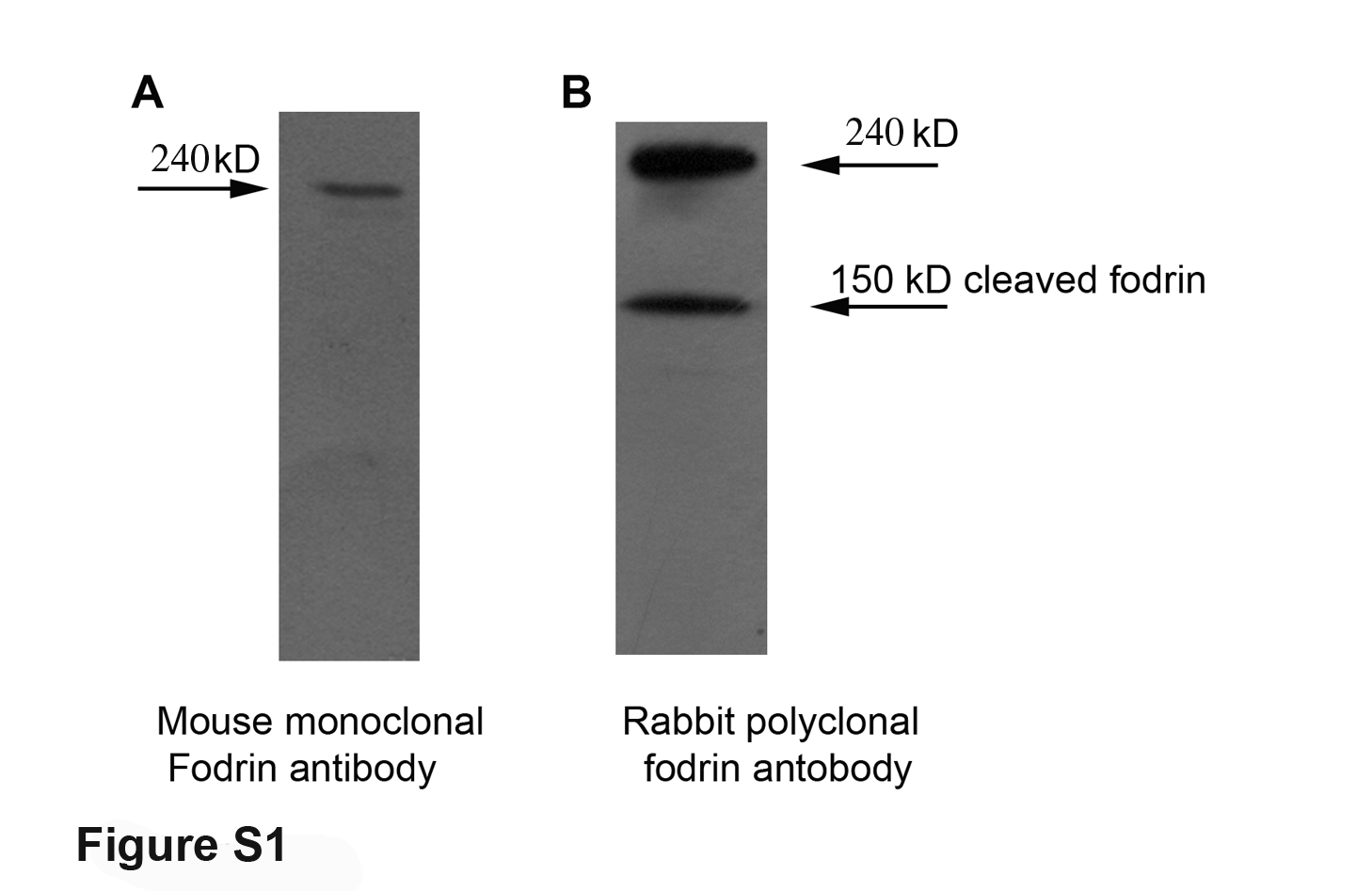

Supplement: Figure S1 — Purified γ-TuRC was treated with antibodies against α-fodrin at 1∶500 dilution and developed by respective secondary antibodies. (TIF) [file pone.0076613.s001.tif]

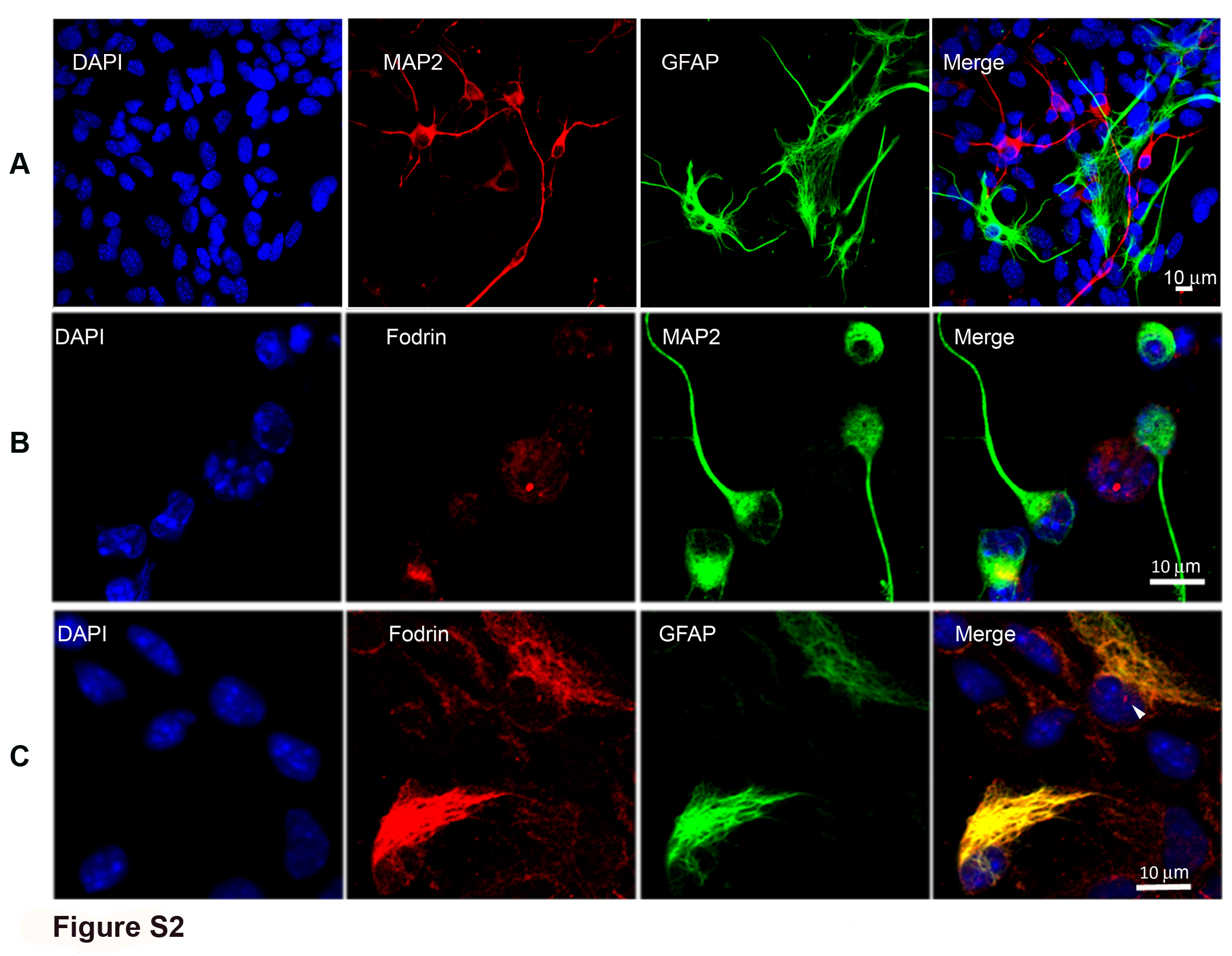

Supplement: Figure S2 — Localization pattern of MAP2 (mature neuronal marker), GFAP (glial marker) and fodrin in primary cells. Cells were isolated from 18 days old mouse embryonic brain and cultured in appropriate media as described in the methods section. Cells were imaged after immunostaining with A: GFAP (green), MAP2 (red); B: MAP2 (green), α-fodrin (red) for mature neurons and C: GFAP (green), α-fodrin (red) for glial cells. DAPI (blue) was used for nuclear staining. (TIF) [file pone.0076613.s002.tif]

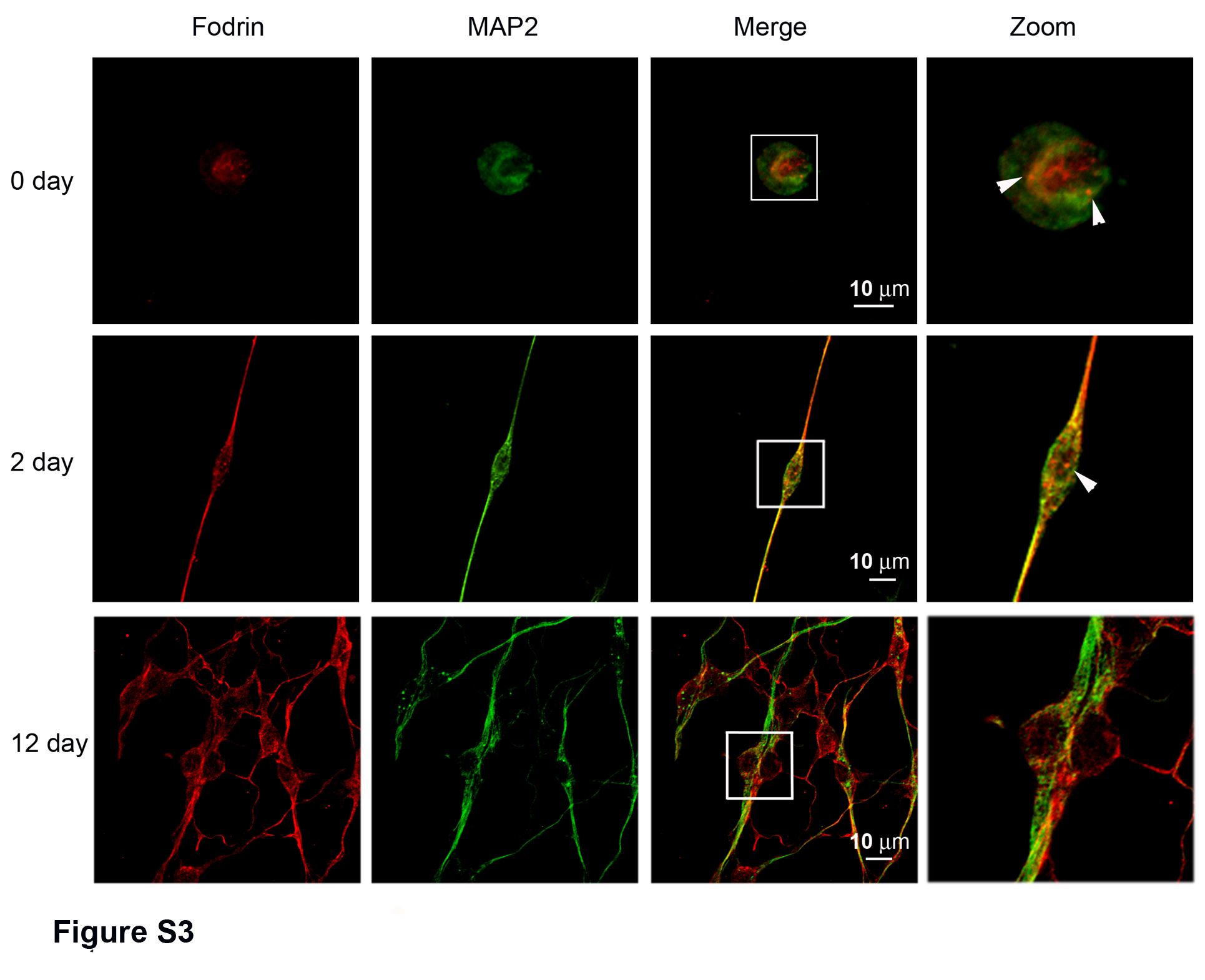

Supplement: Figure S3 — Localization of α-fodrin and MAP2 in primary neurons at different stages. Cells were isolated from E18 mouse embryo and were maintained in neuronal cell enrichment media. Panels show fodrin and MAP2 localization in undifferentiated (0 day), early (2nd day) and late stages of differentiation (12th day). Immunostaining was done with α-fodrin (red) and MAP2 (green). Area of the cells in the boxes is magnified and shown in merge. Arrowheads mark the presence of fodrin on 0 day and 2nd day. (TIF) [file pone.0076613.s003.tif]

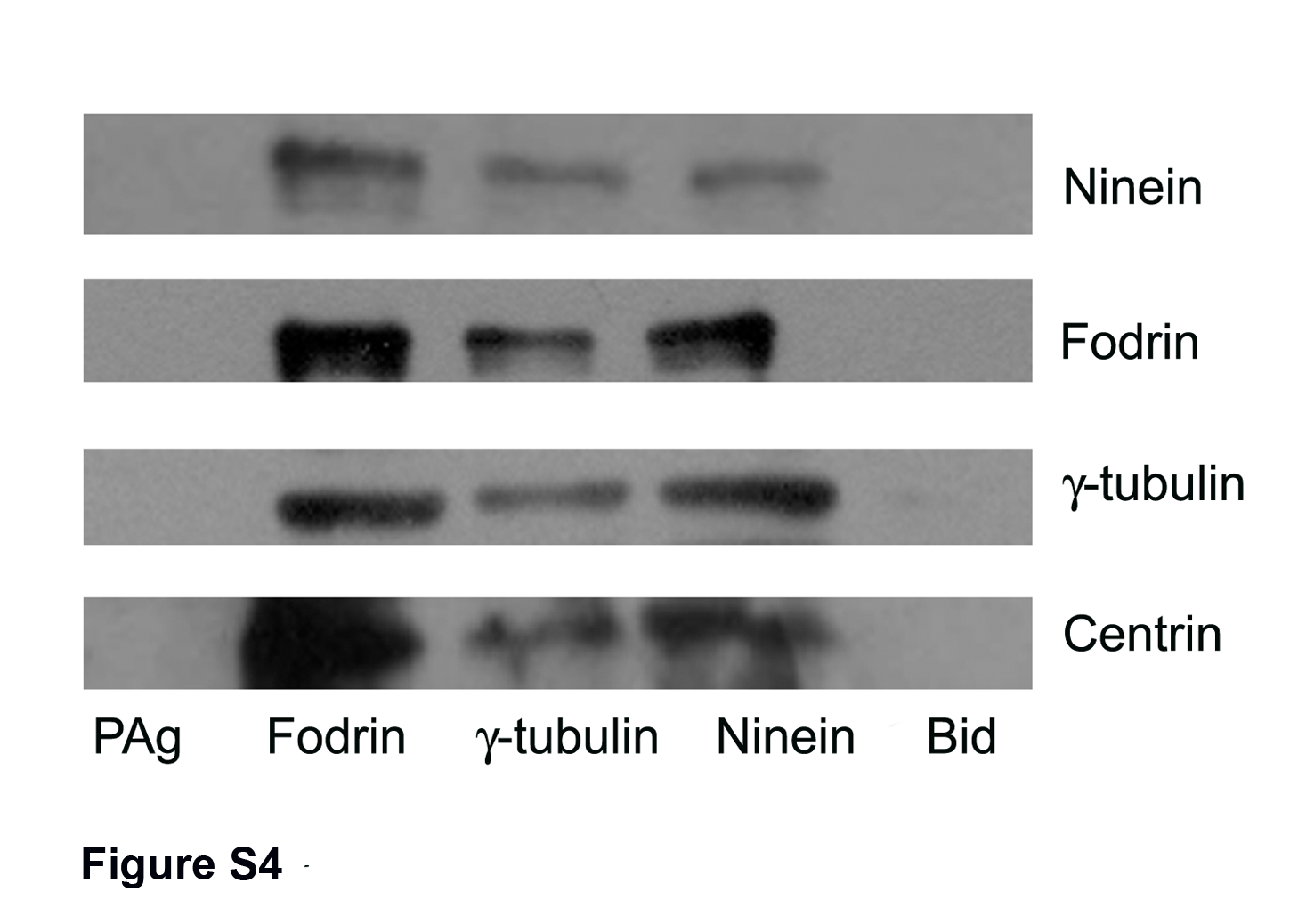

Supplement: Figure S4 — Immunoprecipitation of centrosome fraction: The centrosome fraction (50%) purified by sucrose density gradient was immunoprecipitated with anti ninein, anti γ-tubulin and anti α-fodrin antibodies. Immuno pulldown with PAg and anti Bid antibody were used as negative controls. Western blot was performed with antibodies against ninein, γ-tubulin and centrin in 1∶1000 dilution and α-fodrin in 1∶500 dilution. (TIF) [file pone.0076613.s004.tif]

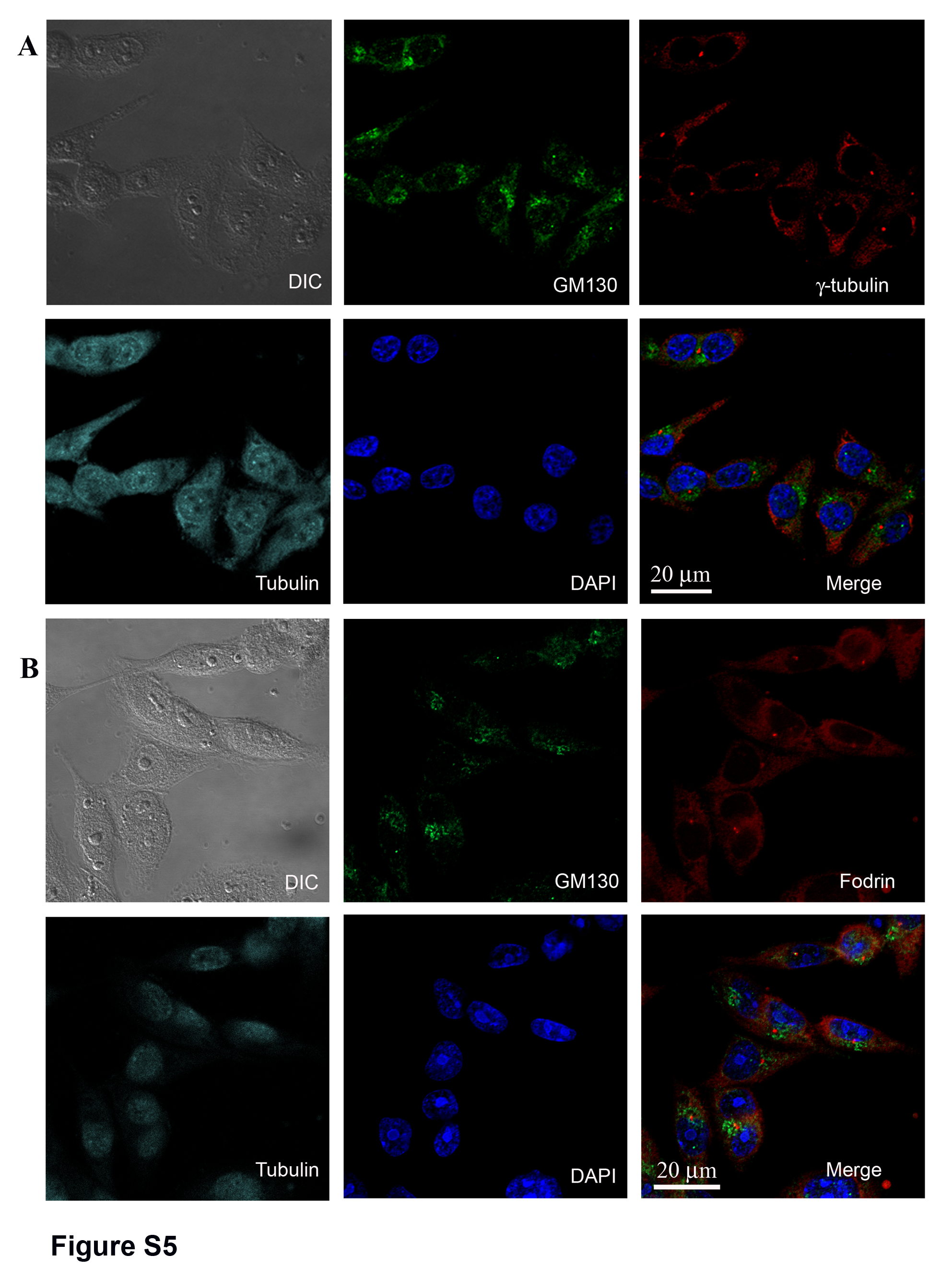

Supplement: Figure S5 — Localization of γ-tubulin or fodrin in golgi apparatus. IMR32 cells were immunostained for A: DAPI, tubulin (cyan), γ-tubulin (red) and golgi matrix protein GM130 (green); B: DAPI, tubulin (cyan), fodrin (red) and golgi matrix protein GM130 (green). Primary and secondary antibodies were used in 1∶200 and 1∶500 respectively. Cells were maintained in DMEM containing 5% FBS with antibiotics. (TIF) [file pone.0076613.s005.tif]
